# Focal Radiation Therapy Combined with 4-1BB Activation and CTLA-4 Blockade Yields Long-Term Survival and a Protective Antigen-Specific Memory Response in a Murine Glioma Model

**DOI:** 10.1371/journal.pone.0101764

**Published:** 2014-07-11

**Authors:** Zineb Belcaid, Jillian A. Phallen, Jing Zeng, Alfred P. See, Dimitrios Mathios, Chelsea Gottschalk, Sarah Nicholas, Meghan Kellett, Jacob Ruzevick, Christopher Jackson, Emilia Albesiano, Nicholas M. Durham, Xiaobu Ye, Phuoc T. Tran, Betty Tyler, John W. Wong, Henry Brem, Drew M. Pardoll, Charles G. Drake, Michael Lim

**Affiliations:** 1 Department of Neurosurgery, Johns Hopkins University School of Medicine, Baltimore, Maryland, United States of America; 2 Department of Radiation Oncology and Molecular Radiation Sciences, Johns Hopkins University School of Medicine, Baltimore, Maryland, United States of America; 3 Department of Oncology and Medicine, Johns Hopkins University School of Medicine, Baltimore, Maryland, United States of America; 4 Departments of Oncology, Ophthalmology, and Biomedical Engineering, Johns Hopkins University School of Medicine, Baltimore, Maryland, United States of America; City of Hope, United States of America

## Abstract

**Background:**

Glioblastoma (GBM) is the most common malignant brain tumor in adults and is associated with a poor prognosis. Cytotoxic T lymphocyte antigen -4 (CTLA-4) blocking antibodies have demonstrated an ability to generate robust antitumor immune responses against a variety of solid tumors. 4-1BB (CD137) is expressed by activated T lymphocytes and served as a co-stimulatory signal, which promotes cytotoxic function. Here, we evaluate a combination immunotherapy regimen involving 4-1BB activation, CTLA-4 blockade, and focal radiation therapy in an immune-competent intracranial GBM model.

**Methods:**

GL261-luciferace cells were stereotactically implanted in the striatum of C57BL/6 mice. Mice were treated with a triple therapy regimen consisted of 4-1BB agonist antibodies, CTLA-4 blocking antibodies, and focal radiation therapy using a small animal radiation research platform and mice were followed for survival. Numbers of brain-infiltrating lymphocytes were analyzed by FACS analysis. CD4 or CD8 depleting antibodies were administered to determine the relative contribution of T helper and cytotoxic T cells in this regimen. To evaluate the ability of this immunotherapy to generate an antigen-specific memory response, long-term survivors were re-challenged with GL261 glioma en B16 melanoma flank tumors.

**Results:**

Mice treated with triple therapy had increased survival compared to mice treated with focal radiation therapy and immunotherapy with 4-1BB activation and CTLA-4 blockade. Animals treated with triple therapy exhibited at least 50% long-term tumor free survival. Treatment with triple therapy resulted in a higher density of CD4^+^ and CD8^+^ tumor infiltrating lymphocytes. Mechanistically, depletion of CD4^+^ T cells abrogated the antitumor efficacy of triple therapy, while depletion of CD8^+^ T cells had no effect on the treatment response.

**Conclusion:**

Combination therapy with 4-1BB activation and CTLA-4 blockade in the setting of focal radiation therapy improves survival in an orthotopic mouse model of glioma by a CD4^+^ T cell dependent mechanism and generates antigen-specific memory.

## Introduction

The prognosis for patients with glioblastoma (GBM) remains poor despite treatment with surgical resection followed by adjuvant radiotherapy and the addition of temozolomide [1], [2]. Immune checkpoint inhibitors have emerged as a promising strategy in cancer immunotherapy. Immune checkpoints are a class of cell surface molecules expressed by activated T and B lymphocytes. Upon engaging their ligands, immune checkpoints inhibit proliferation and activity of immune cells thereby protecting against autoimmunity [3]. Studies and clinical trials of immunotherapy for GBM pointed out the immunosuppressive influence of the GBM microenvironment as a significant hurdle, however, GBM infiltrating immune cells have been found to express immune checkpoint molecules [4], [5]. Blocking these immunosuppressive mechanisms while generating a strong antitumor response is an intuitive strategy for cancer immunotherapy. A variety of tools are now available to test this strategy empirically and move these agents into clinical trials [5].

Our group recently published results demonstrating that PD-1 blockade, in combination with stereotactic radiation therapy resulted in a durable, long-term survival in GL261 bearing mice [6]. Antibodies against co-stimulatory molecules, such as 4-1BB (CD137) and Cytotoxic T-Lymphocyte Antigen 4 (CTLA-4, CD152) have the potential to enhance immune responses and produce anti-tumor immunity [7]–[10]. 4-1BB is expressed on activated T cells and engagement of 4-1BB with its ligand drives proliferation of CD8^+^ T cells, increased pro-inflammatory cytokine production and plays an essential role in the formation of long-lived memory cytotoxic T cells [9], [11], [12]. CTLA-4 signaling impairs the capacity of T cells to proliferate and to produce pro-inflammatory cytokines [13]. Blockade of CTLA-4 removes these suppressive signals and allows antigen-specific T cells to expand and perform their effector functions [7]. Ipilimumab, a human monoclonal antibody that blocks CTLA-4, has been approved by the FDA for first line treatment of advanced melanoma. In phase III trials, ipilimumab improved survival in patients with metastatic melanoma and produced a durable anti-tumor memory response [14], [15]. Ipilimumab has also been shown to induce regression of melanoma brain metastases [16] and may be potentiated by radiation therapy [17]. However, some patients treated with ipilimumab suffered from severe immune-related adverse events, which was consistent with the proposed mechanism of CTLA-4 blockade [14]. An approach to overcome this burden is to combine CTLA-4 blockade with 4-1BB activation: both individual antibodies cause inflammation to selective organs, however, a combination of the two antibodies increased cancer immunity while reducing inflammation and autoimmune effects [18].

To bolster the anti-tumor immunity created by the monoclonal antibodies anti-CTLA-4 and anti-4-1BB, our group investigated the effects of radiation on glioma treatment as well. Radiation therapy has the potential to augment immune responses against central nervous system tumors [19], [20]. Furthermore, cancer cells destroyed by radiation therapy are considered to be a source of tumor associated antigens that can be processed by professional antigen presenting cells [21].

We investigated the use of focal radiation therapy in addition to anti-4-1BB and anti-CTLA-4 immunotherapy as a combination strategy in an orthotopic, preclinical model of malignant glioma. We hypothesized that radiation therapy followed by 4-1BB activation and CTLA-4 blockade produces an effective and durable anti-tumor response against intracranial GL261 gliomas.

## Materials and Methods

### Ethics statement

Animal procedures were performed in accordance with institutional protocols and approved by the Johns Hopkins University Animal Care and Use Committee (protocol # MO09M395).

### Mice

Six- to eight-week old female C57BL/6 mice (Harlan) were maintained under pathogen-free conditions at Johns Hopkins University.

### Cells and reagents

Luciferase-expressing cell lines, GL261-luciferase (GL261-luc) glioma, and B16-luc melanoma cells were produced as previously described [6]. GL261-luc glioma cells and B16-luc melanoma cells were cultured in DMEM supplemented with 10% fetal bovine serum and 1% penicillin-streptomycin at 37°C in a 5% CO_2_ and 95% humidified air atmosphere. Anti-CTLA-4 mAb producing hybridoma 4F10 was purchased from American Type Culture Collection and the anti-4-1BB hybridoma 2A was kindly provided by Dr. Lieping Chen (Yale University). Both antibodies were purified from supernatant by a protein G column. Hamster and rat IgG to serve as control antibodies were purchased from Rockland Immunochemicals Inc. CD4-FITC and CD8-PE antibodies were used for FACS analysis (BD-Pharmingen). Anti-CD4 and anti-CD8 rat antibodies were used for *in vivo* depletion studies (BioXcell).

### Treatment protocol

The orthotopic glioma model was established as previously described [6]. Animals were stratified into treatment groups on day 7 following intracranial implantation based on tumor burden as determined by bioluminescent imaging. For those mice treated with focal radiation therapy, a single fraction of radiation at a dose of 10 Gy was delivered using a 3 mm collimator on day 10 following intracranial implantation using the Small Animal Radiation Research Platform (SARRP) [22]. With the built-in micro-CT scanner we identified the burr hole, which served as the coordinate for delivery of radiation [23]. In those mice treated with anti-4-1BB mAb, 200 µg of anti-4-1BB antibodies was dosed via intra-peritoneal injection on days 11, 14 and 17 following intracranial implantation. In mice treated with anti-CTLA-4 mAb, 800 µg of anti-CTLA-4 antibodies was dosed via intra-peritoneal injection on days 11, 17 and 23. Controls in all treatment groups received rat and hamster IgG. For the pilot experiments in which treatment with a single antibody was compared to combination therapy with SRS and antibody therapy, five animals were used per treatment group and this experiment was performed once. In the CTLA-4 timing experiments, eight animals were used per treatment group and these experiments were repeated twice. The triple therapy experiments were repeated three times and a total of 18 animals per treatment group were used. Animals were observed three times a week for signs of lethargy such as weight loss, hunched position and epilepsy. In order to reduce suffering when these symptoms occurred, animals were euthanized with cervical dislocation after i.p. injections with a ketamine/xylazine anesthetic. Survival time was recorded and long-term survivors were defined as animals surviving longer than 3 times the median survival of non-treated animals.

### Analysis of tumor infiltrating CD4^+^ and CD8^+^ T cells

Three mice from each treatment group and an additional three naïve mice were randomly selected at day 18 following intracranial implantation and euthanized. Brains and cervical lymph nodes were harvested from each mouse separately and processed into single cell suspensions in RPMI. The cells were filtered through a 40 µm nylon cell strainer, centrifuged at 1000 rpm, and resuspended in 4 mL of 30% Percoll. The cells were overlaid onto a Percoll gradient (30%/37%/60%) and centrifuged at 1200 rpm for 20 minutes. Lymphocytes were collected from the 37%/60% interface and washed twice in PBS. The cells were stained with anti-CD4 and anti-CD8 antibodies and then fixed. Stained cells were analyzed on a FACS Calibur flow cytometer (BD Biosciences) and data analysis was performed with FlowJo software (TreeStar, Ashland OR).

### Depletion of CD4^+^ and CD8+ T cell subsets *in vivo*

Mice were implanted with GL261-luc cells as described above and on day 7 stratified based on bioluminescence into four treatment groups. On days 5, 6 and 7 mice from the CD4 and CD8 depletion groups received 200 µg of anti-CD4 and anti-CD8 antibody respectively in order to achieve *in vivo* depletion. Treatment was administered as described in the triple therapy arm above. Mice received depletion antibodies every 7 days to maintain the depleted condition. Response to *in vivo* depletion of CD4^+^ and CD8^+^ T cells was recorded as a change in survival time compared to the non-depleted mice receiving triple therapy.

### GL261 and B16 flank tumors

On day 100 after intracranial implantation, long-term survivors from the triple therapy group and naïve (non-tumor bearing) mice were challenged by subcutaneous injection in both hind limbs with 10^6^ GL261-luc cells in 100 µL of mixed PBS and Matrigel (BD Biosciences) in a 1∶1 ratio. A second set of long-term survivors from both the triple therapy group and double antibody group were challenged subcutaneously with 10^6^ GL261-luc cells in the left flank and 10^5^ B16-luc cells in the right flank. Tumor growth was measured every 2 to 3 days using calipers and tumor volumes were calculated in three dimensions using the formula: 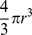. Animals were observed for an additional 50 days or euthanized when tumor volumes reached approximately 1000 mm^3^ in each flank.

### Statistical analysis

Survival was analyzed with the log-rank Mantel-Cox test. Comparison of infiltrating T cells in the brains and lymph nodes was performed with the Student's t-test. All statistical analyses were performed using GraphPad Prism 5 (GraphPad Software Inc., La Jolla, CA).

## Results

### Focal radiation therapy combined with CTLA-4 blockade prolonged survival in established intracranial GL261 tumors

**Figure 1:**
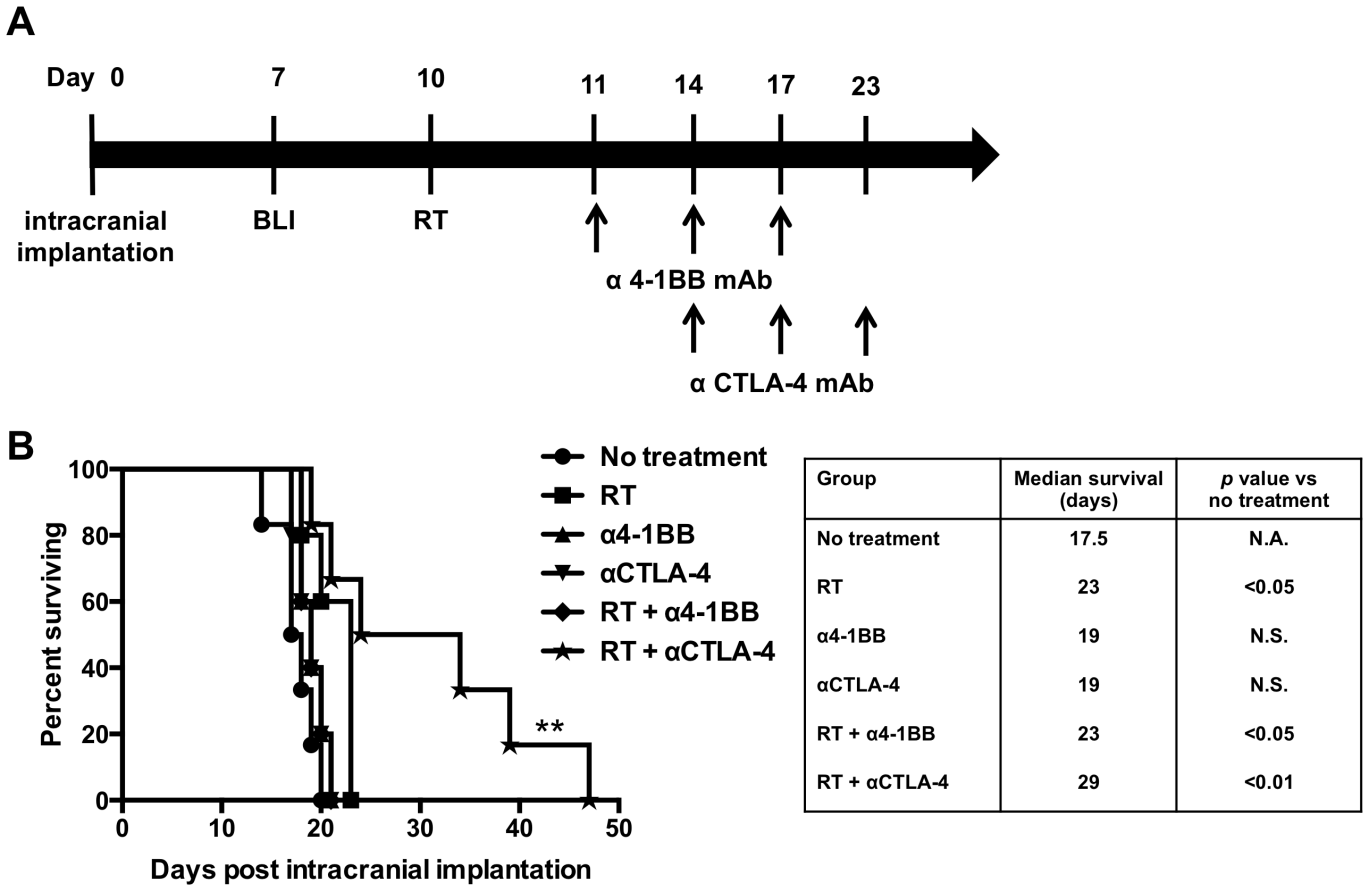
Focal radiation therapy combined with CTLA-4 blockade prolonged survival in established intracranial GL261 tumors. A) Schematic of the treatment protocol for combination therapy with RT and 4-1BB activation or CTLA-4 blockade. B) Kaplan Meier survival curves for single immunotherapy with anti-4-1BB antibodies and combination therapy with RT and 4-1BB activation or CTLA-4 blockade (*n* = 5–6 mice/group). Single immunotherapy with anti-4-1BB antibodies did not extend median survival (*p* = 0.16 vs. untreated mice). Combination treatment with RT and 4-1BB activation extended median survival times significantly (*p*<0.05 vs. untreated mice), however, this result was not significant when compared to treatment with focal RT alone (*p* = 0.99). CTLA-4 blockade alone did not extend median survival in treated animals, however, trended towards significance (*p* = 0.11 vs. controls). Combination therapy with RT and anti-CTLA-4 antibodies prolonged overall survival significantly compared to either treatment modality alone (*p* = 0.045 vs. RT; one-sided Log-Rank test). *P* values were calculated with the Log-Rank test.

Our first aim focused on determining the efficacy of combination therapy with focal RT and 4-1BB activation or CTLA-4 blockade. We proceeded with the treatment protocol shown in Figure 1A. Treatment with 4-1BB agonist antibodies alone did not enhance survival when compared to untreated mice (*p* = 0.16). The addition of 4-1BB agonist antibodies to RT extended survival significantly compared to untreated mice (*p*<0.05). However, this combination did not significantly differ from treatment with focal RT alone (Fig. 1B; *p* = 0.99). Combination therapy with RT and CTLA-4 blockade improved survival in our glioma model. Animals treated with anti-CTLA-4 antibodies alone did not extend survival in comparison with untreated animals (*p* = 0.11; one-sided Log-rank test). Combination therapy with RT and CTLA-4 blockade resulted in a significantly prolonged survival time than untreated animals (*p*<0.01) and when compared with focal RT alone (*p*<0.05; one-sided Log-rank test). Mice treated with the combination of RT and CTLA-4 blockade had a median survival of 29 days, and 50% of mice treated with this combination where still alive after 30 days whereas all mice from the anti-CTLA-4 antibody alone and focal RT alone group died before day 30 after intracranial implantation (figure 1B). These results show that combination therapy with focal RT and anti-CTLA-4 antibodies prolongs overall survival in treated mice.

### Optimal timing of treatment with anti-CTLA-4 antibodies does not influence therapeutic efficacy

**Figure 2:**
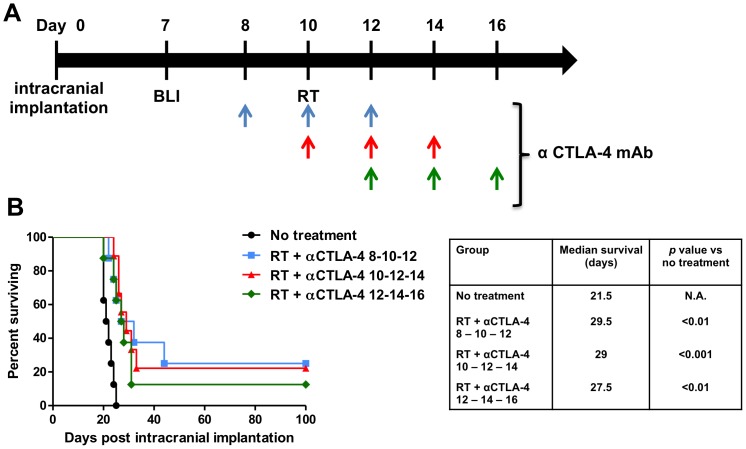
The role of timing in efficacy of anti-CTLA-4 antibody therapy. A) Schematic of the treatment protocol for the CTLA-4 blockade timing experiment. Please note that all treated animals received RT on day 10 and three antibody injections in three different timing schedules (colored arrows). B) Combination therapy with RT and CTLA-4 blockade results in long-term survival compared to controls (*p*<0.01), however, there was no significant difference in treatment efficacy between the three combination therapy groups. *P* values were calculated with the Log-Rank test.

We next assessed whether timing of CTLA-4 blockade influenced treatment efficacy (Fig. 2A). Animals treated with focal RT first on day 10 followed with CTLA-4 blockade on days 12, 14 and 15, had a significantly longer survival time of 27.5 days compared to untreated animals (Fig. 2B; *p*<0.01). When CTLA-4 blockade was administered simultaneously with RT on day 10 followed with injections on days 12 and 14, median survival extended from 21.5 days to 29 days (*p*<0.001 vs. untreated). CTLA-4 blockade starting two days prior to RT on day 8, and followed with injections on days 10 and 12, resulted in a median survival of 29.5 days (*p*<0.01 vs. untreated). Survival times of all three RT and CTLA-4 blockade combination groups were comparable. This suggests that the treatment efficacy of the combination treatment (RT and CTLA-4 blockade) is independent of the sequence of the two therapeutic components. Moreover, these results confirm that combination therapy with focal RT and CTLA-4 blockade prolong overall survival resulting in long-term tumor free survival in 25% of treated mice from the earlier time points group (day 8–10–12), 22% long-term survival in treated mice from the middle time points group (day 10–12–14), and 12.5% long-term survival in treated mice from the later time points group (day 12–14–16).

### Combining stereotactic radiosurgery with 4-1BB activation and CTLA-4 blockade results in long-term survival

**Figure 3:**
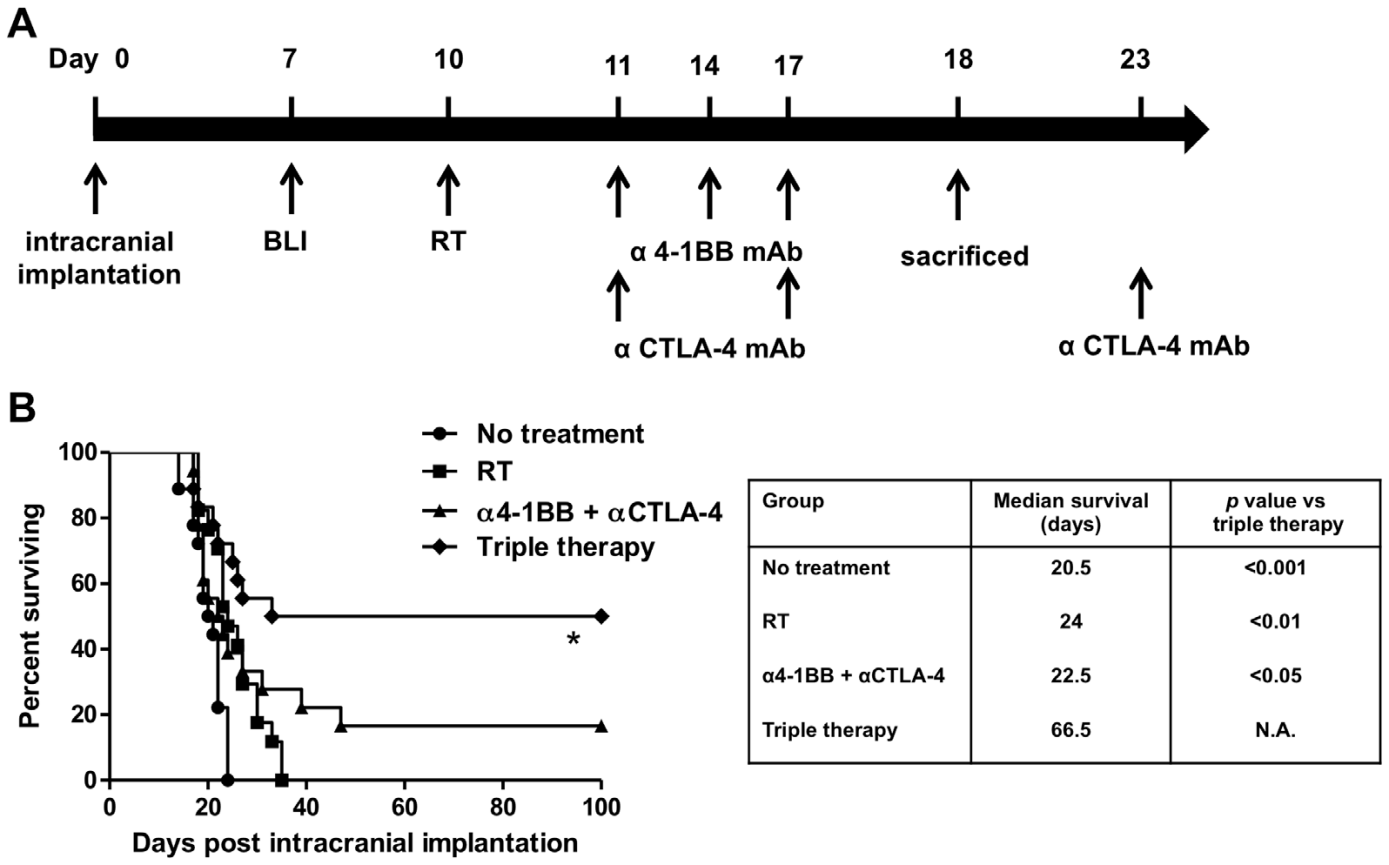
Combining focal radiation therapy with anti-4-1BB and anti-CTLA-4 antibodies results in long-term survival. A) Schematic of the treatment protocol for triple therapy. B) Kaplan Meier survival curves for double immunotherapy and triple therapy (*n* = 18/group). Treatment with triple therapy was superior to double immunotherapy with anti-4-1BB and anti-CTLA-4 antibodies (*p*<0.05), focal RT alone (*p*<0.01) and untreated mice (*p*<0.001). Furthermore, triple therapy results in long-term survival in at least 50% of treated animals, whereas immunotherapy with both 4-1BB activation and CTLA-4 blockade produced long-term survival in 3/18 mice (16.7%). Data from three repeated experiments with similar results was pooled into single Kaplan Meier curves. *P* values were calculated with the Log-Rank test.

After confirming that combination therapy with RT and 4-1BB activation or CTLA-4 blockade extends median survival, we proceeded with combining all three modalities into a “triple therapy” (Fig. 3A). Immunotherapy with the combination treatment of anti-4-1BB and anti-CTLA-4 antibodies resulted in a significantly higher median survival of 23 days instead of 21.5 days when untreated (Fig. 3B; *p*<0.05). Furthermore, where treatment with RT failed to produce long-term survival, immunotherapy with anti-4-1BB and anti-CTLA-4 antibodies produced a long-term tumor free survival in 3 out of 18 mice (16.7%). Triple therapy with RT, 4-1BB activation and CTLA-4 blockade extended the median survival from 24 days when treated with focal RT to 67 days (*p*<0.05 vs. all other treatment modalities) with 50% of the mice going on to become long-term survivors.

### Triple therapy leads to increased CD4^+^ and CD8^+^ infiltrating lymphocytes in the brain

**Figure 4:**
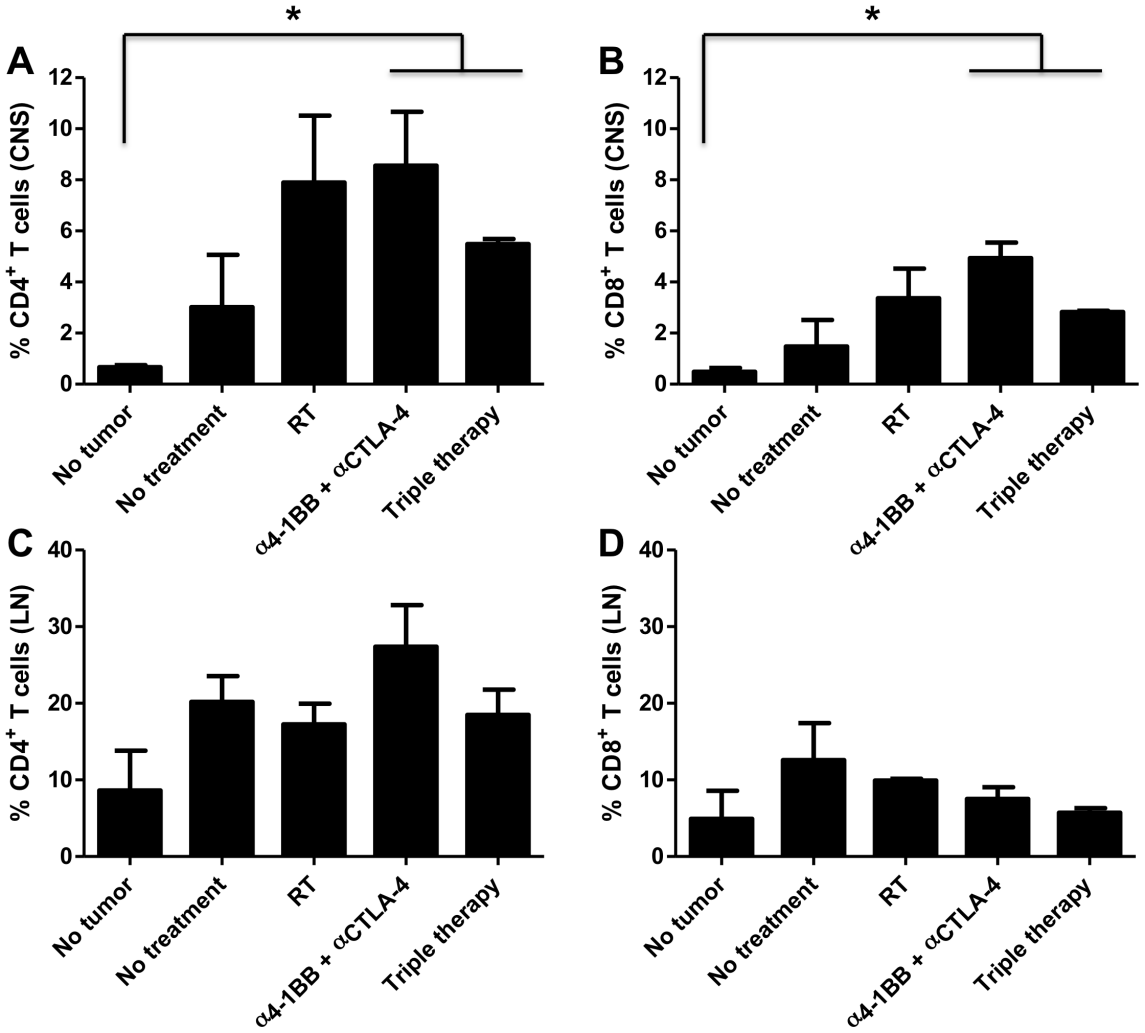
Triple therapy leads to increased CD4^+^ and CD8^+^ infiltrating lymphocytes in the brain. A, B) Influx of CD4^+^ and CD8^+^ tumor infiltrating lymphocytes respectively was higher in brains of animals treated with immunotherapy with 4-1BB activation and CTLA-4 blockade and triple therapy compared to brains from non-tumor bearing mice (*p*<0.05). C, D) Cervical lymph nodes from naïve mice showed a higher baseline of CD4^+^ and CD8^+^ T cells which resulted in comparable densities of T cells between all groups. Error bars represent standard error of the mean (SEM) and p values were calculated with the student t-test.

After establishing the significance of the triple therapy regimen *in vivo*, we quantified infiltrating T cells in brains and cervical lymph nodes harvested from animals in each treatment arm. We found significantly higher numbers of infiltrating CD4^+^ T cells in the brains of mice treated with the combination of anti-4-1BB and anti-CTLA-4 antibodies as well as the mice treated with triple therapy when compared to brains of non-tumor bearing mice (Fig. 4A; *p*<0.05). We also found similar results when we looked at infiltrating CD8^+^ T cells; the density of CD8^+^ T cells from these groups was significantly higher than that of non-tumor bearing mice (Fig. 4B; *p*<0.05). In order to assess the immune modulation in the periphery, we collected and analyzed CD4^+^ and CD8^+^ T lymphocyte populations from the draining (cervical) lymph nodes from mice in each treatment group. We observed no significant differences in the percentages of CD4^+^ and CD8^+^ lymphocytes when looking specifically at the cervical lymph nodes (Fig. 4C and 4D). At day 18 post intracranial tumor implantation, we thus did not observe any changes in T cell populations in the periphery for any treatment group, however, the primary tumor site did exhibit significant changes in both CD4^+^ and CD8^+^ T cell percentages in the triple treatment group as well as the anti-4-1BB and anti-CTLA-4 double treatment group. These findings indicate that differences in lymphocyte modulation exist between the primary tumor site within the brain and the draining lymph nodes in the periphery.

### The anti-tumor activity of triple therapy is CD4^+^ T cell dependent

**Figure 5:**
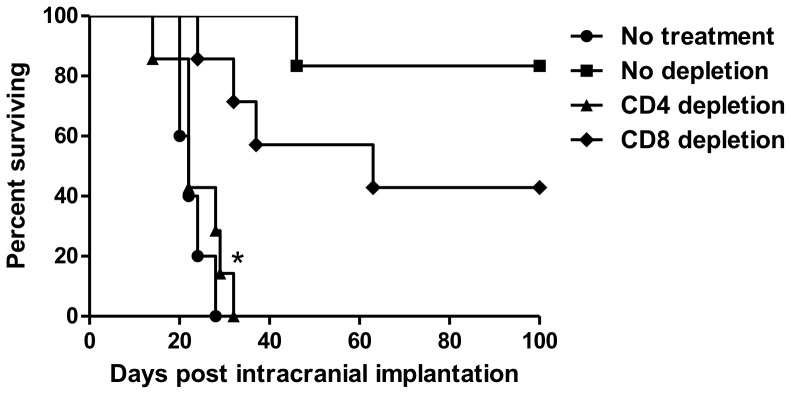
The anti-tumor activity of triple therapy is CD4^+^ T cell dependent. Kaplan Meier survival curves for untreated animals, triple therapy (no depletion), triple therapy with depleted CD4^+^ T cells and triple therapy with depleted CD8^+^ T cells (*n* = 5–7 mice/group). CD4^+^ T cell depletion abolishes the survival benefit of triple therapy (*p*<0.001). Depletion of CD8^+^ T cells did not interfere with the efficacy of triple therapy and resulted in long-term survival.

To determine which subset of immune cells contributes to the anti-tumor effect elicited by triple therapy, CD4^+^ and CD8^+^ T cells were depleted with monoclonal antibodies in mice that subsequently received treatment with triple therapy. Depletion of CD4^+^ T cells completely abolished the anti-tumor activity of triple therapy (*p*<0.001) and median survival times were comparable to untreated mice (Fig. 5). In contrast, mice with depleted CD8^+^ T cells survived significantly longer than untreated mice, as well as significantly longer than mice in the CD4^+^ depletion group (*p*<0.01). Moreover, depletion of CD8^+^ T cells also resulted in 43% of mice exhibiting long-term survival making this group comparable to the non-depleted triple therapy group. These data suggest that long-term survival due to triple therapy is primarily CD4^+^ T cell dependent.

### Treatment with triple therapy results in a glioma-specific protective memory response

**Figure 6:**
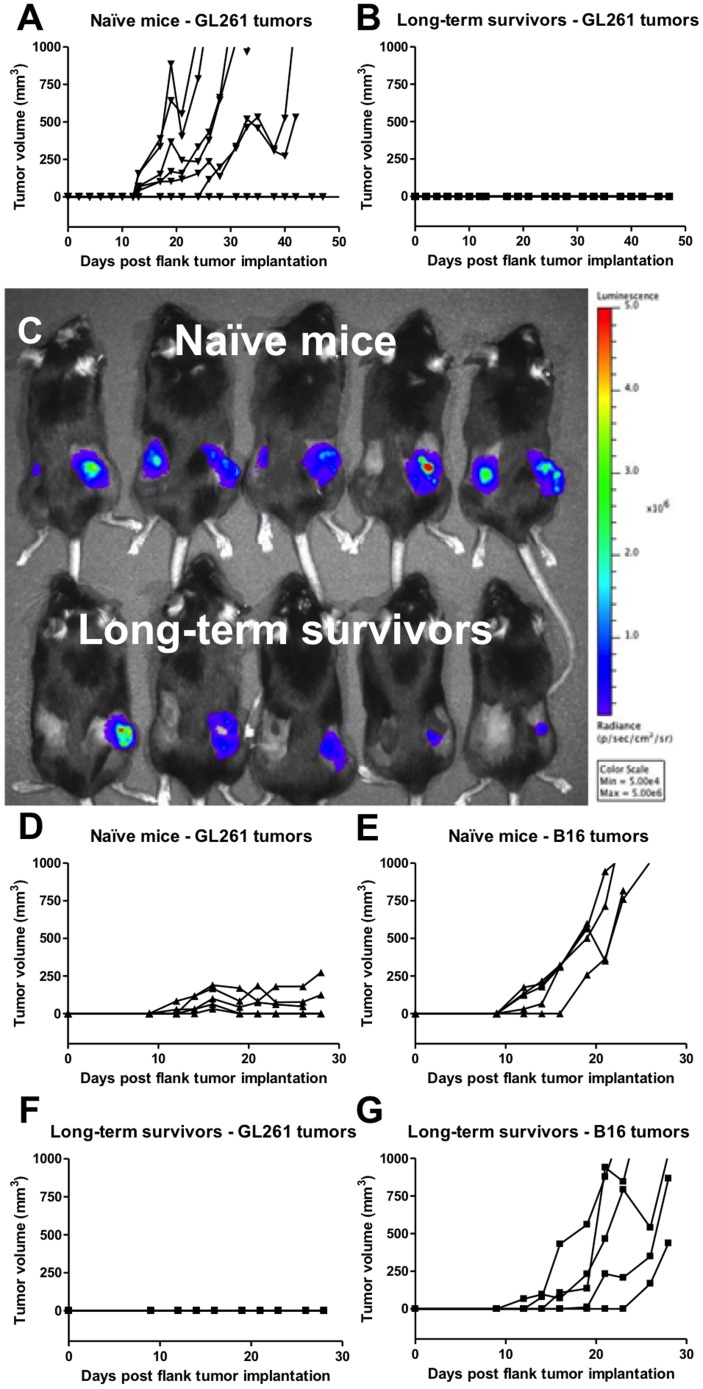
Treatment with triple therapy results in a glioma-specific memory response. A) Naïve mice (*n* = 4/group) challenged with GL261 cells in both flanks formed flank tumors in 6/8 flanks. All control animals formed GL261 flank tumors; two animals formed bilateral flank tumors and two animals formed unilateral flank tumors. B) Long-term survivors (*n* = 3/group) developed long-lasting immunity to GL261 tumors and successfully rejected flank tumor formation in 6/6 flanks. Mice were observed for 50 days. Each line represents tumor growth in one flank. C) Bioluminescent imaging data on day 21 after flank tumor implantation for an experiment where naïve mice (top row) and long-term survivors (bottom row) were inoculated subcutaneously with 10^6^ GL261-luc cells in the *left* flank and 10^5^ B16-luc cells in the *right* flank. D) Naïve mice had progressive GL261 and E) B16 flank tumor growth. Note that B16 tumors grew faster in comparison to GL261 tumors. F) Long-term survivors established a protective memory response and rejected GL261 flank tumor growth. G) Long-term survivors established a glioma-specific memory response which did not affect the formation of B16 melanoma flank tumors.

To evaluate whether long-term survivors had developed a protective anti-tumor memory response, we challenged them with a subcutaneous flank injection of GL261 cells at day 100 after their first intracranial implantation. Figure 6A displays the tumor growth measurements for each flank tumor (n = 8) in naïve animals (n = 4); in all four animals, tumors had progressive growth by day 28. However, all of the long-term survivors (*n* = 3; 6 flank tumors), that had successfully rejected the intracranially implanted tumor in the first round of experimentation, did not develop flank tumors (Fig. 6B). Next, we developed a model to determine whether the systemic anti-tumor memory response in long-term survivors is specific for GL261 glioma tumors only or is also responsive to other types of tumors. We compared GL261 glioma and B16 melanoma tumor growth in long-term survivors and in naïve mice. GL261 cells were implanted subcutaneously into the left flank, while B16 cells were implanted into the right flank. Figure 6C shows bioluminescent imaging data on day 21 after GL261 and B16 flank tumor implantation in both naïve mice and long-term survivors. Naïve mice grew both tumor types resulting in palpable nodules on both flanks (Fig. 6D and 6E), whereas long-term survivors grew only B16 tumors with no evidence of palpable GL261 tumors (Fig. 6F and 6G). The observed difference between absent GL261 glioma and active B16 melanoma growth, therefore, indicates a glioma-specific anti-tumor memory response.

## Discussion

In our preclinical study, we have shown that triple therapy combining RT with anti-4-1BB and anti-CTLA-4 antibodies confers long-term survival in a murine GL261 glioma model. The long-term survivors exhibit a glioma-specific memory response. Furthermore, we have shown that triple therapy is primarily CD4^+^ T cell dependent.

Immunotherapy has been widely tested in both pre-clinical studies and clinical trials as a treatment for multiple cancer types. These studies have shown promising results when immunotherapy has been tested against less aggressive tumor types, but fail to achieve equally robust anti-tumor responses when utilized against some of the most malignant tumor models. More specifically, immunotherapy combining 4-1BB activation and CTLA-4 blockade resulted in tumor rejection in 100% of treated animals in a murine RM-1 prostate model [24]. This double therapy also resulted in significant tumor rejection in established MC38 colon tumors. While the combination of anti-4-1BB and anti-CTLA-4 antibodies produced tumor rejection in these two commonly less aggressive tumor types, the same therapy failed to produce tumor growth delay in the highly aggressive B16 melanoma model [25]. Combination immunotherapies have also been tested as a treatment in murine models of glioma: the combination of GVAX followed by CTLA-4 blockade resulted in 50% long-term survival in a murine model of intracranial glioma, whereas CTLA-4 blockade alone did not improve survival in established tumors [26]. Conversely, murine intracranial SMA-560 gliomas treated with anti-CTLA-4 antibodies as a monotherapy elicited long-term survival in 80% of treated animals [27]. Though this particular study found a survival benefit resulting from the administration of one immune modulating agent, the vast majority of immunotherapy studies implicate combination therapy as more efficacious in fighting tumor growth. We found that when both antibodies were combined with radiation, treatment not only resulted in prolonged survival but also produced a durable tumor free long-term survival.

The synergy of immune modulating agents has been well documented, and the specific combination of 4-1BB activation and CTLA-4 blockade offsets any toxicity incurred by using one agent on its own. The addition of 4-1BB activation to CTLA-4 blockade reduced the severe immune related adverse events that result from CTLA-4 blockade in a preclinical study [18]. Treatment with the human monoclonal CTLA-4 blocking antibody ipilimumab resulted in severe immune related adverse events in a clinical trial that made termination of treatment inevitable [14]. It seems that the occurrence of these severe immune related events was related to the mechanism of CTLA-4 blockade. Treatment with nivolumab, a human monoclonal antibody blocking PD-1, resulted in a milder toxicity profile [28]. Furthermore, the addition of nivolumab to ipilimumab reduced the rate of severe immune related events related to CTLA-4 blockade remarkably, which affirms the benefits of combining monoclonal antibodies [29].

The addition of radiation to immunotherapy provides an environment conducive to immune mediated killing of tumor cells in order to activate effective antigen presentation. This strategy prevents cancer cells from escaping immune recognition [19], [20], [30]. While radiotherapy is part of the standard treatment regimen for malignant glioma, the high number of fractions may actually hinder the ability of the immune system to target and kill cancer cells [31]. We previously published results suggested that focal radiation could augment an immune response [6]. We used a unique small animal irradiator to deliver precise radiation making our approach an attractive regimen for translation into the clinic [22], [23]. Although our results show that treatment with double immunotherapy – without focal RT – resulted in tumor free survival in 17% of treated animals, triple therapy remained superior by consistent tumor rejection in at least 50% in all animals. Interestingly, we found that the treated animals either had a complete objective response to triple therapy that resulted in the formation of a protective memory or failed to respond to treatment. One possible explanation could come from the fact that there is likely variability in the sizes of each animal's tumor (hence some could have margins outside of the treatment field) and perhaps a certain amount of the tumor needed to be exposed to radiation to elicit the protective response. In addition, the addition of focal RT to CTLA-4 blockade resulted in long-term survivors. As a result, our findings suggest that radiation is synergistic when combined with immunotherapy.

In addition, we found that the timing of antibody treatment with regard to RT did not influence treatment efficacy [32]. One point to note was that the timing of the CTLA-4 antibody administration was different due to logistics. In the triple therapy protocol, anti-CTLA-4 antibodies were administered in a six day interval in order to reduce the risk of auto-immunity when combined with anti-4-1BB antibodies. For the CTLA-4 timing experiment, the interval of antibody administration was shortened from six days to two days in order to assess the timing relative to RT delivery. CTLA-4 blockade administered on three separate time schedules relative to RT did not differ in result suggesting that treatment efficacy is independent of a specific therapeutic window. Surprisingly, when CTLA-4 blockade was given in three dosages of 800 µg in a two-day interval – instead of the six day interval employed in our triple therapy protocol – the treatment was able to produce tumor free long-term survival.

We sought to examine the mechanism by which the triple therapy affects T cell infiltration in the brain. It is known that CTLA-4 blockade removes suppressive signals and allows expansion of tumor-specific T cells, in particular of CD4^+^ effectors [7]. On the other hand, 4-1BB activation is known to co-stimulate CD8^+^ T cells and increases their proliferation and survival [12]. We hypothesized that triple therapy will result in higher CD4^+^ and CD8^+^ tumor infiltrating lymphocytes. When compared to non-tumor bearing mice, our results show that there is indeed an influx of CD4^+^ and CD8^+^ T cells into the brains from the groups in which immunotherapy with 4-1BB activation and CTLA-4 blockade is employed. The depletion study provided information about which of these two T cell subsets determines the efficacy of triple therapy. Interestingly, animals treated with triple therapy with CD8^+^ T cell depletion responded to treatment and resulted in 43% long-term survival whereas depletion of CD4^+^ T cells completely abolished the effect of triple therapy. This is surprising considering a previously published study stating that the tumor-eradicating effect of 4-1BB activation with CTLA-4 blockade in a MC38 colon cancer model is primarily CD8^+^ T cell dependent [18]. Furthermore, our recently published study showed that combination therapy with focal RT and PD-1 blockade was primarily CD8^+^ T cell dependent and that depletion of CD4^+^ T cells did not interfere with treatment efficacy [6]. We hypothesized that triple therapy would be CD8^+^ T cell dependent as well, however, our results suggest that triple therapy is CD4^+^ T cell-dependent and, more surprisingly, CD8^+^ T cell-independent. The precise mechanism remains unknown.

We have found that our triple therapy regimen has the potential to eliminate tumor cells and confer a survival advantage to over 50% of animals treated with the regimen. One additional desirable outcome of using immunotherapy is to develop a memory response to tumor cells such that highly aggressive tumors such as GBM do not have a chance to recur. To assess the establishment of a protective tumor response, long-term survivors were re-challenged with GL261 cells in both flanks and were capable of rejecting glioma flank tumor formation. In contrast, when long-term survivors were peripherally injected with B16 melanoma cells, flank tumors were formed. It is known that gliomas and melanomas share melanoma-associated antigens like trp2 and gp100 [33], [34], however, our results suggest a glioma-specific memory response. This finding is consistent with the concept that the anti-tumor memory response in this model involves glioma-specific antigens. Future work is needed to identify these antigens and evaluate their specificity.

## Conclusion

Treatment with focal radiation therapy followed by double immunotherapy with anti-4-1BB and anti-CTLA-4 antibodies significantly extends survival and, more importantly, produces tumor free long-term survival. Triple therapy is dependent on an intact CD4^+^ T cell compartment, whereas depletion of CD8^+^ T cells did not abrogate treatment efficacy. Furthermore, triple therapy provides for a durable antigen-specific memory response.
